# The Mechanism of Saccade Motor Pattern Generation Investigated by a Large-Scale Spiking Neuron Model of the Superior Colliculus

**DOI:** 10.1371/journal.pone.0057134

**Published:** 2013-02-19

**Authors:** Jan Morén, Tomohiro Shibata, Kenji Doya

**Affiliations:** 1 Graduate School of Informatics, Kyoto University, Kyoto, Japan; 2 Mathematical Informatics Laboratory, Nara Institute of Science and Technology, Nara, Japan; 3 Neural Computation Unit, Okinawa Institute of Science and Technology, Okinawa, Japan; Barrow Neurological Institute, United States of America

## Abstract

The subcortical saccade-generating system consists of the retina, superior colliculus, cerebellum and brainstem motoneuron areas. The superior colliculus is the site of sensory-motor convergence within this basic visuomotor loop preserved throughout the vertebrates. While the system has been extensively studied, there are still several outstanding questions regarding how and where the saccade eye movement profile is generated and the contribution of respective parts within this system. Here we construct a spiking neuron model of the whole intermediate layer of the superior colliculus based on the latest anatomy and physiology data. The model consists of conductance-based spiking neurons with quasi-visual, burst, buildup, local inhibitory, and deep layer inhibitory neurons. The visual input is given from the superficial superior colliculus and the burst neurons send the output to the brainstem oculomotor nuclei. Gating input from the basal ganglia and an integral feedback from the reticular formation are also included.

We implement the model in the NEST simulator and show that the activity profile of bursting neurons can be reproduced by a combination of NMDA-type and cholinergic excitatory synaptic inputs and integrative inhibitory feedback. The model shows that the spreading neural activity observed in vivo can keep track of the collicular output over time and reset the system at the end of a saccade through activation of deep layer inhibitory neurons. We identify the model parameters according to neural recording data and show that the resulting model recreates the saccade size-velocity curves known as the saccadic main sequence in behavioral studies. The present model is consistent with theories that the superior colliculus takes a principal role in generating the temporal profiles of saccadic eye movements, rather than just specifying the end points of eye movements.

## Introduction

The mammalian visuomotor system is one of the best studied model systems for elucidating the computational principles of movement control and their neural implementation mechanisms. Within the visuomotor system, the superior colliculus (SC) plays a primary role in directing the gaze by eye and head movements by integrating multiple sensory and cognitive inputs [1]–[3]. There have been a variety of models of the SC in visuomotor control from abstract linear system models to detailed models incorporating the anatomical and physiological reality [4]. Recent studies have revealed that the number and the temporal profile of the spikes of the SC output neurons are precisely regulated to attain a desired eye movement [5]. Computational models of the SC to date, however, mostly consider population firing rates and continuous neural field approximation and do not allow investigating the spike-level computation and neurobiological mechanisms.

The aim of this study is to construct a realistic spiking neuron model of the SC that allows us to bring together the behavioral functions of eye movements, the anatomy and physiology of the SC circuit, and the biophysical properties of the SC neurons.

The SC is composed of the superficial, intermediate, and deep layers. The superficial SC receives visual input from the retina and from cortical visual areas [6]. The intermediate SC receives visual input from the superficial SC directly and through the parabigeminalis [7], [8] and also receives auditory and somatosensory inputs. The intermediate SC is assumed to evaluate the short-term saliency of the sensory features [2], [9]. The intermediate SC representation of saccade targets is retinotopic, and the bursting activity of its output neurons is relayed to the pons and the midbrain areas that generate horizontal and vertical eye and head muscle activation, respectively [10], [11].

A major question about the function of the SC is whether it just specifies the location of a saccadic eye movement or is further involved in shaping of the dynamic pattern of eye movements [5]. By building a spiking-neuron model of the SC based on anatomical and physiological data, we aim to test whether it is possible to reproduce temporal and spatial response profiles of SC neurons that are consistent with the dynamic features of saccadic eye movement.

**Figure 1:**
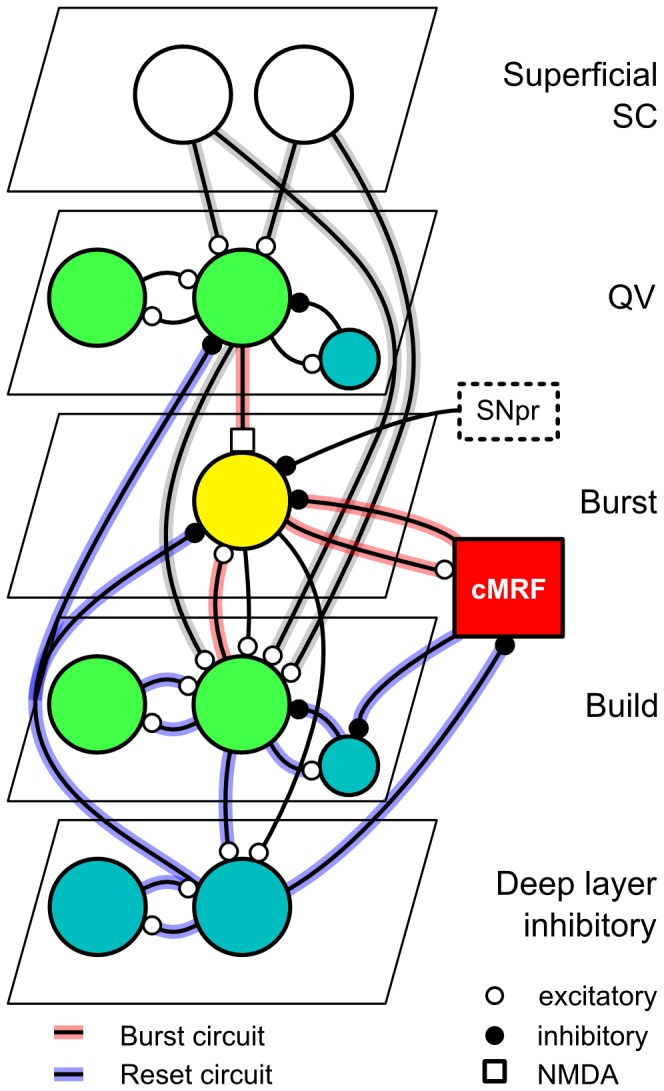
The model circuit. Each neuron model layer is spatially extended and organized in the same relative order as in the neural substrate. Feedback connections to the same node indicate layer intraconnections. Primary functional circuits are indicated by coloured connections. Red connections: Burst neuron burst profile circuit. Blue connections: Spreading inhibition and system reset. Grey shaded connections: inputs common to both circuits. See materials and Methods for further details.

We first review the key anatomical and physiological features of the SC circuit and present the model structure (see figure 1), followed by the neuron model and the general model parameters. We then test the features of NMDA-type synapses and describe how to tune the SC model to behavioral data. We finally show the model response to simulated visual inputs and recreate the saccade main-sequence behavior.

### Anatomy and physiology of the superior colliculus

The superficial SC consists of two interconnected layers and two major cell types – wide-field and narrow-field receptive cells – that project to the intermediate areas [12]. The intermediate and deep SC consists of cells with multiple types of activity, although they are morphologically similar [13]. Excitatory neurons in the intermediate layer are classified into quasi-visual (QV), burst, and buildup neurons [4]. Inhibitory interneurons are located both in the intermediate and deep layers [14], [15].

Burst neurons send their output to the eye movement control nuclei in the brain stem. They act as regular spiking neurons when stimulated directly but exhibit bursting behaviour when they receive inputs from the superficial layer [12] through the quasivisual and buildup neurons [8], [16]. Burst neurons have NMDA-type glutamatergic inputs as well as cholinergic inputs [17]. SC burst neurons are not intrinsically bursting, have no local excitatory interconnections that could facilitate bursting behaviour, and NMDA receptor inactivation inhibits bursting but not regular firing of burst neurons in the rat. The membrane potential-dependent characteristics of the NMDA receptor is a likely mechanism for the bursting behavior [18].

Buildup neurons exhibit spreading activation during a saccade [19]–[21], though the purpose is still unclear [22]–[24]. The proximal mechanism is most likely to be through short-range interconnections [16], [25].

In addition to local inhibitory inputs from within the intermediate SC, burst neurons also receive an extrinsic feedback loop from the saccade motor-related areas such as the central mesencephalic reticular formation (cMRF) that regulates the saccade magnitude and movement profile [26], [27] by counteracting a local inhibitory mechanism in the intermediate and deep SC [28]. The superior colliculus is thus part of the saccade generation feedback loop [29], [30]. The substantia nigra pars reticulata (SNpr) in the basal ganglia tonically inhibits the intermediate SC output neurons [31].

Burst neurons in the rostral pole generate microsaccades during fixation in a manner similar to the saccade-generation circuitry in the caudal SC [32]. The differential function is mediated primarily through differences in the downstream connections, with fixation-time rostral inhibition of saccades through downstream omnipause neurons (OPN)[33].

There is considerable evidence for asymmetric large-scale spreading activation occurring in the intermediate SC during saccades in many animals [21], [23]. While connections in the superficial SC are long-range, both excitatory and inhibitory intraconnections within the intermediate SC seem to be spatially limited to 500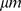 or less [25]. Some have reported intra-collicular long-range connections aside from those from the rostral pole (see cf. [34], [35]). However they are not monosynaptic or they could be the effect of excitation of axons of passage [36]. The spreading activation is not likely to be a direct effect of monosynaptic long-range connection within the intermediate areas. The asymmetry may arise from the complex-logarithmic mapping from the retina to the SC [37], though see [38]. That still leaves open the question both of the functional significance of the spreading activation and of the mechanism by which is it achieved.

### The role of SC in the generation of saccades

While the superior colliculus is implicated in the generation of saccades it is only one area in a large functional network that includes the Frontal Eye Fields and the Cerebellum. It is known that either SC or the FEF alone can generate saccades in the absence of the other in the primate [39]. In the case of SC inactivation animals exhibit a lack of short-latency saccades, suggesting that the SC is mainly concerned with bottom-up, low latency eye movements.

The horizontal and vertical eyeball motoneuron systems need to work in close synchrony in order to generate straight, on-target saccades. The movement initiation time and the movement profile over time both need to be closely coupled [40]. Gisbergen argues that the tight temporal coupling between the horizontal and vertical movement profiles implies that they are likely to be generated from a common source, rather than generated independently for the vertical and horizontal components. As the SC is a final common point for the downstream motor systems, it is one likely point for generating not just the common target signal, but also the movement profile over time.

**Figure 2:**
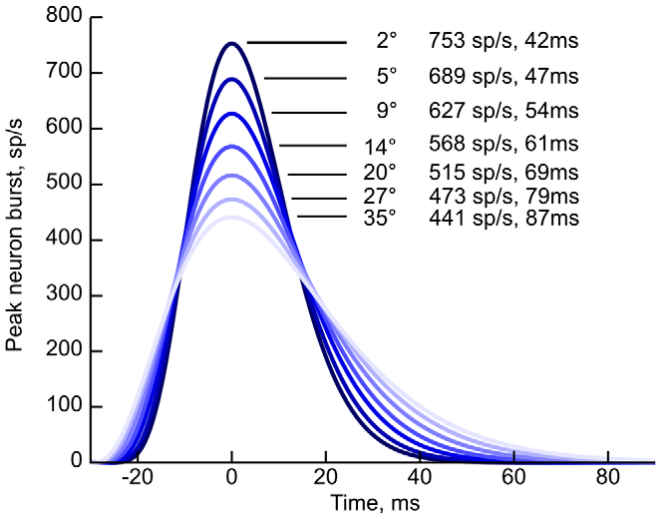
Parametrized burst neuron activation profiles based on monkey data. Curves show peak firing rate and peak-to-end firing time, where the end is taken when the curve drops below 1% of peak. Regenerated from [5].

If the superior colliculus indeed provides a movement profile for the brainstem eye-muscle driving neurons rather than just a target position, the burst cell output rate over time needs to follow a specific profile. Goossens and van Opstal [5] showed that burst neurons in the monkey SC indeed generate output rate profiles that conform to the expected pattern (figure 2). They recorded single burst neurons in monkeys performing saccades, and show that burst neuron activity follow the expected profile. In addition, disruption of saccades show that variation in the SC burst neuron profile pattern and eye movement profile over time is closely correlated.

Goossens and van Opstal [5], [29] also proposed that burst output spikes directly drive the downstream horizontal and vertical saccade-generation systems, with the direction set by the position of the neuron in the collicular map. Total burst spike counts are held approximately constant; the saccade magnitude is instead regulated by the connection strength between SC burst neurons and the downstream systems, with stronger connections eliciting a larger movement per spike.

The Cerebellum is heavily implicated in saccades downstream of the SC and FEF. The oculomotor vermis lesion causes significant dysmetria and large timing and end-goal variations, though saccades still exhibit distance-dependent variation of peak velocity and duration [41]. This suggests that the cerebellum adjusts the timing and magnitude of movements, compensates for errors in the downstream brainstem systems and the motor actuators, but is not the source of the movement profiles themselves [42].

Taken together it is plausible that the target selection and the movement profile is generated upstream of the cerebellum and motoneuron systems; that is, in the superior colliculus as well as in areas related to the FEF. SC burst neuron discharges encode a motor target as well as a time profile that drives brainstem eye-muscle driving neurons.

Our aim is to explore a possible neural circuit that can generate such a collicular burst neuron movement profile over time, subject to physiological and neurological constraints.

We are concerned in this paper with the direct subcortical saccade generation loop as an integrated sensory-motor system. We thus consider only the superior colliculus and leave the interaction with the frontal eye fields aside. This has some biological justification; so-called express saccades are visually triggered saccades with very short latency that are generated in a bottom-up fashion through the retina-SC-motor system loop without the involvement of cortical areas [43]. We currently consider only the saccade signal generation process in the caudal SC. The rostral pole microsaccade circuit in the SC is functionally very similar, but relies on downstream structures for mutual inhibition with the caudal areas. In the absence of rostral pole-initiated fixation-time inhibitory feedback we let the SNpr input mechanism directly and solely control the saccade activation through cessation of tonic inhibition.

Our model aims to replicate the SC of the macaque monkeys. However, when the anatomical of physiological data from macaques are not available, we use the data from other primates, rodents, or rabbits with appropriate scaling. In the following, the observations are from primates unless otherwise mentioned.

## Results

### Model construction

The overall structure of the SC model is illustrated in figure 1. It is composed of the superficial SC, four functional neuron types in the intermediate SC (quasi-visual, bursting, build-up, and deep inhibitory neurons), the cMRF integrator, and a generic inhibitory input marked as SNpr. The neuron types are spatially organized in separate functional layers in the model; the use of “layer” below refers to the model organization rather than to physiological layers in the biological system.

Our proposed burst generation circuit consists of two mechanisms. The first is the activation of burst neurons by NMDA-type input from the QV neurons and the cholinergic input from the buildup neurons and the subsequent recurrent inhibition from the cMRF integrator neurons (figure 1, red connections). This negative feedback circuit creates a rapid burst followed by gradual extinction, with the burst and extinction profile primarily dependent on the strength of the NMDA-synaptic and the cMRF inhibitory inputs to the burst neuron, respectively. The second mechanism is an inhibitory projection to the intermediate areas from deep inhibitory neurons at saccade end triggered by buildup neuron activity (figure 1, blue connections).

*Superficial SC* output is implemented by sets of narrow-field and wide-field neurons. The Gaussian-distributed Poisson spikes with a standard deviation of 0.6 mm representing the visual target location is projected to the superficial units according to a log-polar distribution (eq. 6 and 7). The narrow- and wide-field neurons project to the quasivisual and buildup neurons in the intermediate division. It has been reported that the visual input to the SC has a transient component, but we compensate for the lack of an superficial SC model by using a tonic pulse input.

*Quasivisual (QV) neurons* receive inputs from the superficial SC and from cortical and other sensory areas, and project to the burst and buildup neurons in the intermediate SC. The layer has rostrally shifted intra-layer connections and local inhibitory interneurons that stabilize the layer.

*Buildup neurons* receive inputs from superficial SC and from QV neurons, and project on to the burst neurons. They have mutual short-range excitatory intraconnections, but are reciprocally inhibited by interneurons that limit activity spread. The QV input and the intra-layer connections are rostrally shifted, which we modeled as simple shifted Gaussian projections, though it could be a log-polar convolved Gaussian. This is the mechanism by which buildup neuron layer activation spread becomes directional.

*Burst neurons* are known not to have mutual excitatory interconnections or to be intrinsically bursting. They burst only when receiving both NMDA-type (see Materials and Methods) input from the QV neurons and cholinergic input from buildup neurons. The resulting burst primarily excites ipsilateral eye muscle motoneuron systems, and inhibits the contralateral SC and motoneuron systems. The burst drives the downstream motoneuron systems but is also projected to a set of spike integrator units in the cMRF.

*Deep layer interneurons*, tentatively identified with deep layer inhibitory neurons as found in the rabbit [15] are locally interconnected in order to generate a well-defined burst, and get inputs from burst and buildup neurons. They are silent until the inputs reach a critical level, at which point they fire a burst of inhibitory activity that resets the burst and buildup layers and associated structures such as the cMRF integrator.

*cMRF integrator* integrates the burst neuron inputs at the time scale of 100 ms. This integrated inhibitory signal projects back to the burst neuron layer and to the buildup inhibitory interneurons. This limits the total number of spikes generated in the burst neuron layer, and creates a rapid but controlled drop off in the spike rate. This directly generates the expected saccade main-sequence velocity profile. For our model we use a set of synthetic integrator units in the cMRF rather than a real spiking neuron integrator circuit.

*The substantia nigra pars reticulata (SNpr)* is one source of inhibitory regulation and tonically inhibits output-related structures in the intermediate SC. The inhibition is released when the SNpr is inhibited by the striatum and the currently prepared saccade is triggered. For simplicity we conflate external inhibitory inputs to this in the model. We simulated the SNpr input to SC by a set of Poisson spike generators that are suppressed 100 ms after visual target presentation and reactivated after the offset of the saccade.

The spreading activation occurs among buildup neurons, while burst neuron activity remains confined to a restricted area around the stimulus origin. In order to recreate the experimentally observed rostral activity shift, we approximate the connection asymmetry with rostrally shifted wide-field Gaussian efferent projections and buildup neuron short-range interconnections (see table 1). Reciprocal interneurons restrict buildup-neuron activity, and interneuron inhibition from the cMRF in turn allows buildup neuron activity to spread.

**Table 1:** Model interconnection parameters.

Connection	radius	sd	Wt	ConnP.
Input 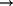 wide	1.5	0.6	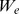	0.07
Input 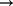 narrow	0.2	–	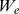	0.25
wide 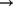 QV	0.2	–	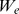	0.25
wide 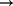 Build	0.2	–		0.25
narrow 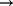 QV	0.2	–	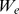	0.25
narrow 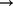 Build	0.2	–	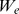	0.25
QV 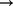 QV	0.5	0.6	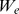	0.175 (1)
QV 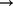 InQV	0.6	0.4	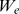	0.25
InQV 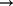 QV	0.6	0.4	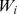	0.25
QV 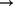 Build	0.5	0.2	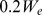	0.25
QV 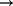 Burst	1.5	0.5	(2)	0.25
Build 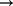 Build	0.5	0.6	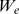	0.175 (1)
Build 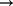 Burst	0.5	0.4	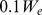	0.25
Build 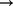 InB	0.6	0.4	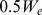	0.25
InB 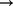 Build	0.6	0.4	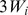	0.25
Build 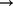 Inhib	2.0	1.0	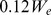	0.05
Burst 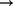 Build	0.5	0.3	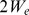	0.25
Burst 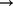 Inhib	0.5	0.1	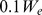	0.025
Burst 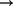 INT	–	–	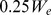 (3)	–
Inhib 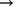 Inhib	0.5	0.4	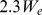	0.25
Inhib 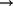 Burst	0.5	0.5	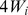	0.25
Inhib 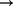 QV	0.5	0.5	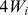	0.25
Inhib 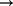 INT	–	–	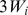	– (3)
INT 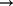 Burst	–	–	(2)	– (3)
INT 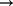 InB	–	–	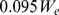	– (3)

Radius: receptive radius, mm; sd: standard deviation, mm; Wt: weight (in terms of synaptic conductance); ConnP: interneuron connection probability.

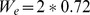
 nS: excitatory synapse; 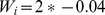 nS: inhibitory synapse;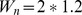 nS: NMDA synapse. Connection delays are 1 ms unless otherwise specified.

input: input source; wide: Wide-field superficial neuron; narrow: Narrow field superficial neuron; QV: Quasivisual neuron; InQV: Quasivisual interneuron; Build: Buildup neuron; InB: Buildup interneuron; Burst: Burst neuron; Inhib: Deep layer inhibitory neuron; INT: cMRF integrator.

(1): interconnections are rostrally shifted by 0.3 mm, and with a 5 ms delay.

(2): Connection weight varies by a saccade angular distance-dependent factor; see Results for details.

(3): Integrator connections have 5 ms delay.

We propose that the amount of activation elicited by the spreading activation keeps track of overall system activity to act as a local shut-down mechanism. As activity increases through the buildup layer it eventually triggers deep layer inhibitory neurons in the deep SC [15] that in turn inhibits collicular areas, resets the cMRF integrator (figure 1, blue connections).

### Role of NMDA-type synapse in burst neuron firing

**Figure 3:**
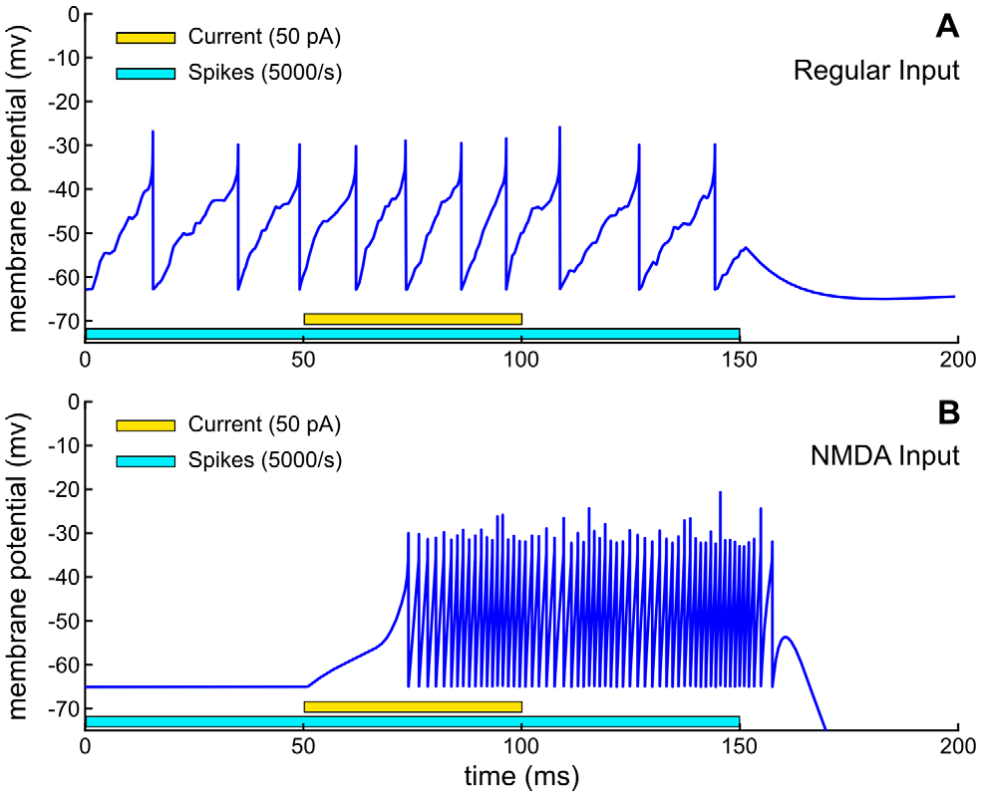
Model neuron response to a Poisson spike train input. Input to a regular excitatory synapse (top) and to an NMDA-type synapse (bottom). A 50 pA input current is added between 50 ms and 100 ms. Model parameters in table 2.

We first investigate the role of NMDA-type synapse in shaping the bursting neuron activity and find appropriate set of parameters to satisfy the constraint on the burst duration and spike counts. The responses to regular and to NMDA-type synaptic inputs are shown in figure 3. The neuron is unresponsive to NMDA-type synaptic input when the neuron is near the resting potential (bottom). When the membrane potential is also raised through a current injection, which mimics the cholinergic synaptic input, the neuron starts bursting due to the voltage-dependent synaptic current (equation 1). The activation sustains even after the current input is removed.

### Parameters for variable burst patterns

In order to find the model parameters to satisfy the output spike patterns shown in Figure 2 while the total spike count is constant [29], we tested how the burst neuron activity profile depends on the NMDA-synaptic weight and the cMRF inhibitory feedback strength.

**Figure 4:**
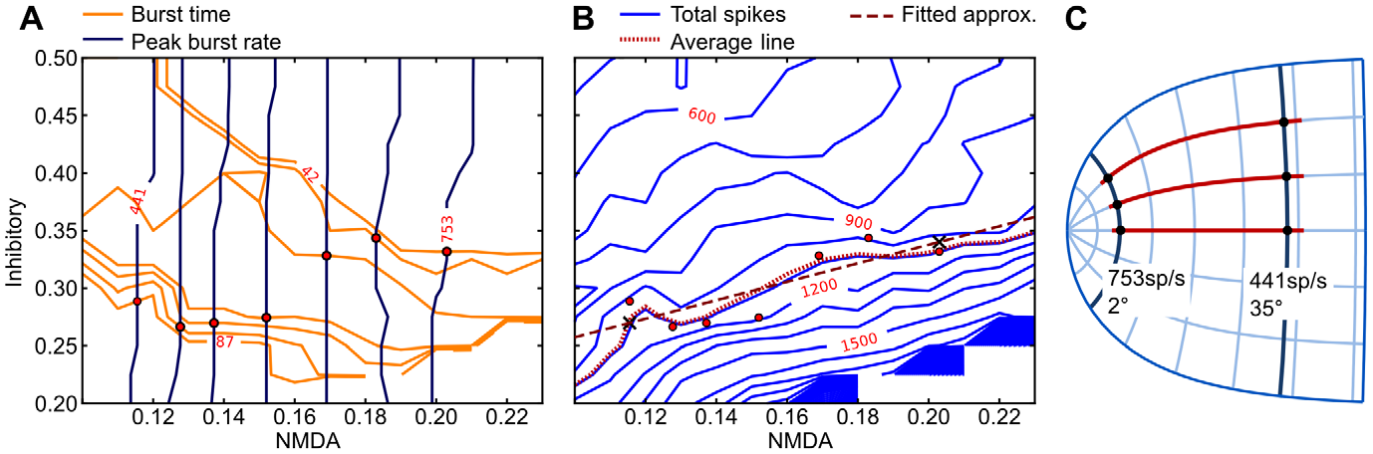
Angle-dependent parameter estimation. A Model peak-to-end burst time (orange contour plot) and equal peak burst rate (blue contour plot) as functions of QV
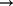
burst NMDA synapse strength (horizontal axis) and cMRF inhibitory feedback strength (vertical axis). Axis values are factors of the nominal NMDA synapse and inhibitory synapse strengths (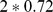
nS, 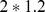
nS) as specified in Table 2. The selected contour line values correspond to the peak-to-end burst times and peak burst rates from figure 2. Red marks show intersections of burst time and burst rate for the same magnitude saccade, and signify the combination of parameters that will give us burst neuron activation corresponding to a saccade of that magnitude.B The total peak-to-end spikes (blue contour lines) as a function of NMDA and inhibitory strength. Burst end is determined when activity drops below 10% of peak activity. Red line: the contour line corresponding to the average emitted spikes for all points from 4A (1088 spikes, red marks). Hashed line: the linear approximation of the selected contour line. Cross marks signify the 35 degree and 2 degree points.C NMDA and inhibitory synapse strength across the SC surface is set according to this linear approximation as the radius from the fovea in SC surface coordinates.

Figure 4A and 4B show the effect of NMDA synaptic strength and inhibitory feedback strength on burst time (A, orange), peak burst rate (A, blue), and burst spike totals (B, blue). The contour plot surface data is generated from model simulations with combinations of parameter values. We want to determine how these two connection parameters should change as a function of saccade distance.

From the parametrized monkey data shown in figure 2 we know the expected peak burst rate and burst time for a sample of angles. The blue and orange contour lines corresponding to these rates and times are shown in figure 4A. Their pairwise intersections, marked as red dots, correspond to the NMDA and inhibitory connection strengths that will generate saccades with peak burst rates and burst times matching this data.

We need to satisfy the additional restriction that total spikes be constant for any angle saccade. In figure 4B we see the total burst spikes as a function of the connection parameters (blue contour lines), together with the intersection points we found in figure 4A. The average burst spike total for these points is 1088 spikes, marked as a red dotted contour line across the surface. We fit this contour line with a straight-line approximation, shown as a wide hashed line. The X-marks show the points corresponding to 2 degrees (right end) and 35 degrees (left end).

We use this linear estimation to let the inhibitory and NMDA parameters vary as a function of radial angular distance in SC surface coordinates. Figure 4C illustrates the points on the SC surface corresponding to 2 degrees and 35 degrees foveal distance. The parameters to the target burst nodes thus vary linearly in proportion to the distance from the fovea on the SC surface.

### Saccade pattern generation

**Figure 5:**
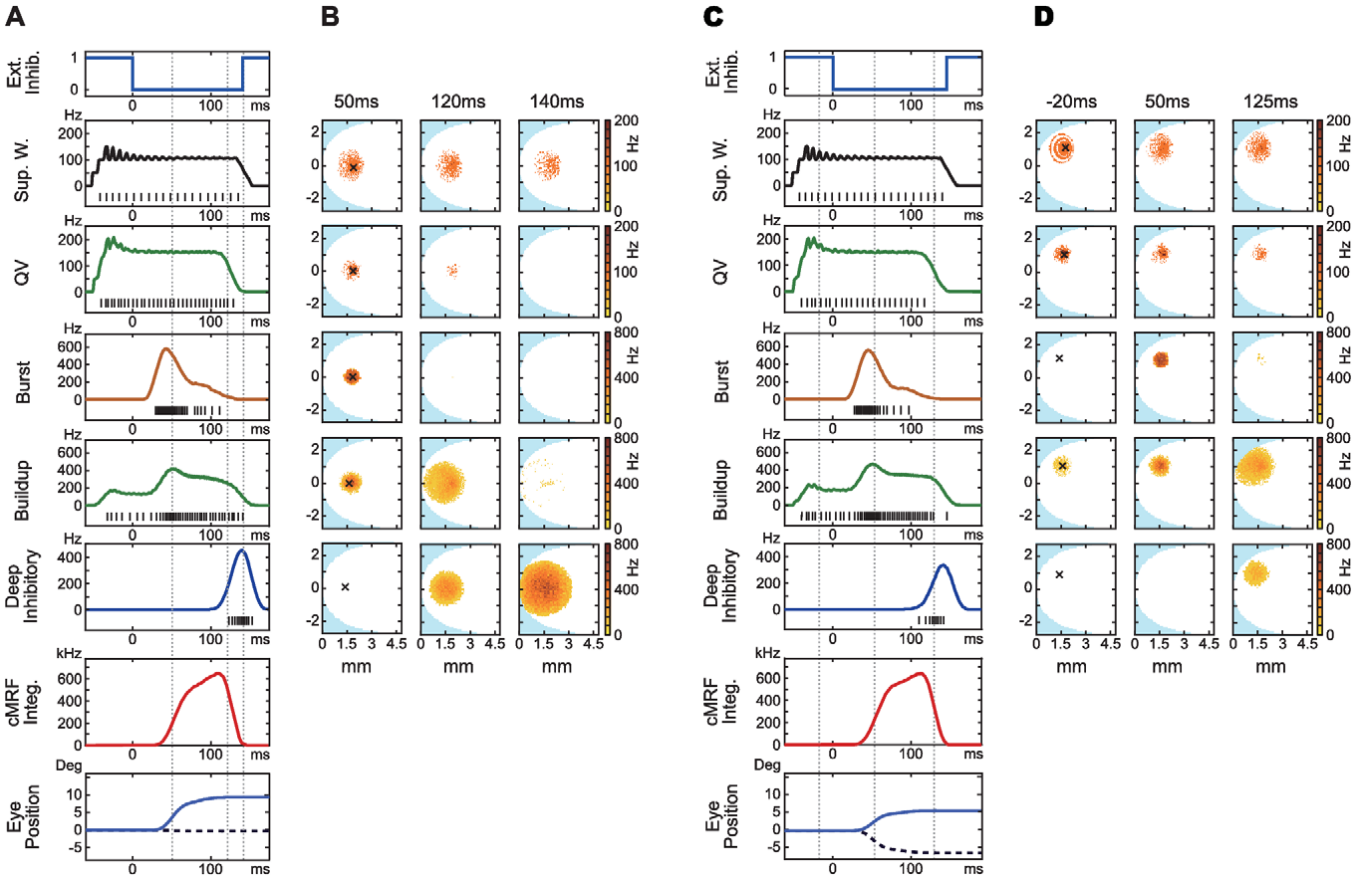
Two saccades. A, B: target input at 9 radius, 0 angle. C,D: target input at 8 radius, 45 downward angle. Times are shown relative to the release of external burst neuron inhibition in both cases.A, C: average firing rate in a 0.1 mm radius around the point of peak activity for each saccade, averaged across ten trials. Black marks: spike trains from the peak neuron in each model layer. From top to bottom: SNpr inhibition; superficial wide-field neurons (wide-field); quasivisual neurons (QV); burst neuron (Burst); buildup neuron (Buildup); deep inhibitory neurons (Deep Inhibitory); cMRF neuron integrator units (cMRF integrators); and estimated horizontal (solid) and vertical (hashed) eye position.B, D: 10 ms snapshots of spatial model layer activity at significant points during the saccades. Times are relative to release of external inhibition. B: burst and buildup peak (50 ms), buildup neuron maximum spread (120 ms), deep inhibitory reset (140 ms). D: Preparatory activity during external inhibition (−20 ms), burst and buildup peak (50 ms) buildup maximum spread (125 ms). One point corresponds to one neuron, single trial. Crosses mark peak activity position for the saccade in each layer.

Here we report the overall dynamic operation of the network and show that the model can reproduce the major physiological features of the SC and saccadic eye movement. The time course of operation of the entire model is illustrated in figure 5. Two separate saccades with stimuli at 9 radius eccentricity, 0 horizontal angle direction, and 8 radius, 45 angle are presented in figure A–B and C–D respectively. A and C show the averaged activity around the center of activation for each neuron type, while B and D shows 10 ms spatial activity around significant points during the course of a saccade. The corresponding times are marked with vertical lines in A and C. All times are relative to the release of external inhibition at 0 ms.

QV neurons and buildup neurons are activated by narrow- and wide-field neuron input in the superficial SC, and are reciprocally inhibited by their inhibitory interneurons (QV inhibitory interneurons not shown) (5D: −20 ms). When the burst neurons are released from SN inhibition at 0 ms, the burst neurons that receive both cholinergic input from the buildup neurons and NMDA input from the QV neurons will activate strongly (5B,D: 50 ms).

The burst output is projected to the spike integrator in the cMRF. This integrated output is projected back to the burst neurons where it inhibits the bursting activity, giving rise to the skewed burst activity profile. The integrator output also inhibits the inhibitory interneurons for the buildup neurons, where asymmetric intraconnections cause a controlled spread of activation over time. This spreading activation (5B: 120 ms, D: 125 ms) eventually triggers deep inhibitory neurons that in turn inhibits the burst and QV neurons (5B: 140 ms) and resets related areas to finish the saccade. The burst neuron activity causes eye movement with 20 ms latency [44]. The visual input is turned off and the SN inhibition is re-established after the saccade.

### Burst rate and duration relationship

**Figure 6:**
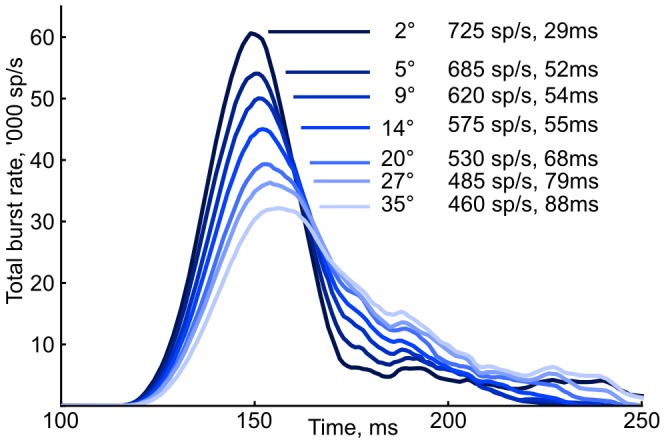
Angle-dependent total burst spike neuron output. Shown for the same angles as in figure 2, rate estimated by 20 ms sliding window, average of ten runs. The burst end is estimated to be when the rate drops below 10% of the peak rate.

The saccade angle-dependent burst profiles is shown in figure 6. The plots and all estimates are based on a sliding 20 ms averaging of ten runs at each point. The resulting profiles reproduce the monkey recording data by [5] as reproduced in figure 2.

### Saccade main sequence

The eye-movement profile – the saccade main sequence – is highly stereotypical and exhibits three distinctive features with the increase in the saccade angle: sub-linear increase in the peak eye movement speed, nearly linear increase in the saccade duration, and skewed velocity profiles with a nearly constant peak time. See [45] for human main sequence data and [46] for the macaque monkey.

**Figure 7:**
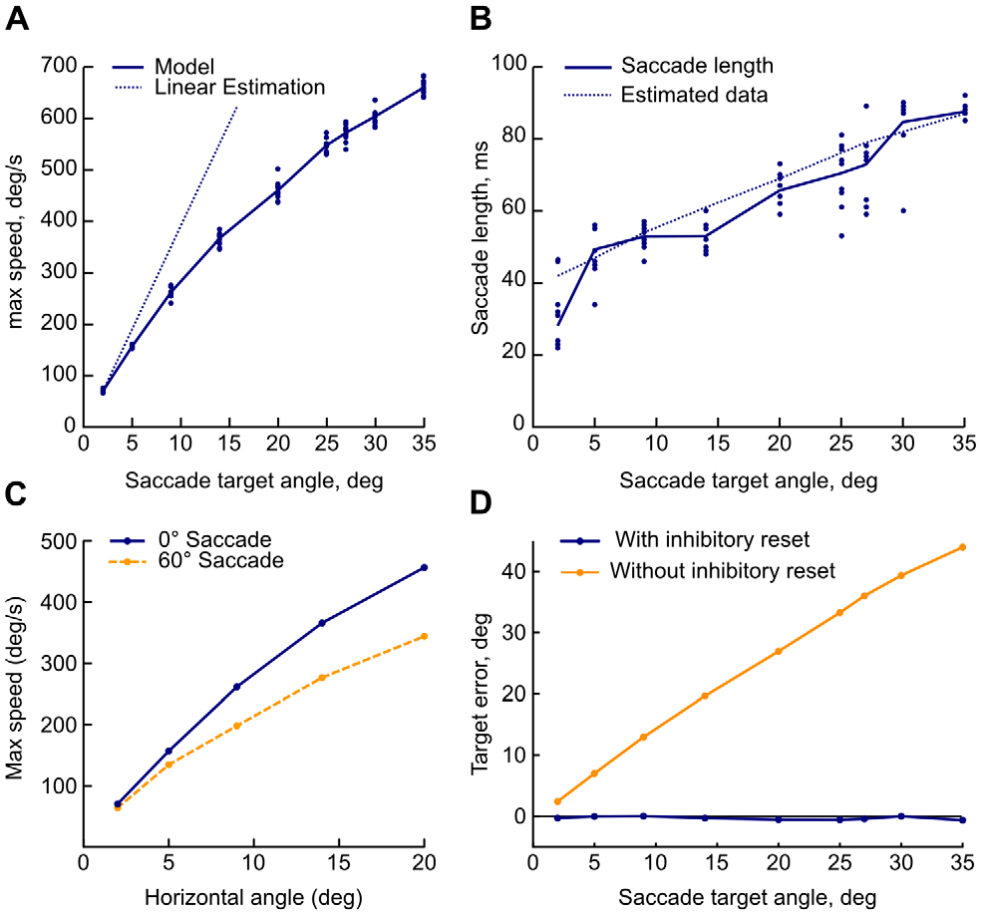
Estimated eye movement profile from model output. A: Peak saccade speed as a function of saccade distance. Circles are individual trials; solid line is the average per simulated distance; and dotted line is the hypothetical linear relation based on the 2 average. B: Saccade time as a function of angular distance. Circles are individual trials; solid line is average per simulated distance; hashed line is parametrized monkey data from [5]. C: Peak saccade speed as function of horizontal distance for 0 and 60 directions. The oblique saccades show the expected drop in peak speed. D: The estimated final eye position discrepancy as a function of target saccade position for the model with (solid) and without (hashed) deep inhibitory neuron feedback, terminated 300 ms after inhibitory release.

Figure 7A shows the predicted eye angle speed generated from the output of our model, under the assumption that the outputs are weighted as above and directly drive the eyeball motoneuron systems. In contrast, the saccade duration varies linearly with saccade angle. Figure 7B shows the model burst neuron activation duration (peak-to-end, solid line) as a function of saccade angle. The hashed line shows the estimated duration from the parametrized monkey data of Goossens and Opstal. The result is expected given the way we set the downstream weights, but validates that we are in fact generating the correct burst profiles.

Figure 7C shows the peak saccade speeds for a straight horizontal saccade (0) and oblique saccade (60) as function of horizontal distance. The oblique saccade exhibits lower peak speed and longer duration (not shown), as observed experimentally [40]. This is a natural consequence of the way we set the NMDA and inhibitory synaptic parameters, based on the radial distance from the fovea rather than the horizontal and vertical components, as described in the previous section.

Figure 7D shows the final estimated position discrepancy of the saccade as a function of saccade target, with the weights and gain specified in earlier sections. The full model (solid line) is consistently close to the intended distance for all tested angles. In order to test the role of the spreading activation of the buildup neuron model layer and subsequent inhibition from the deep layer neurons, we ran a simulation by setting the connection weights from the deep layer inhibitory neurons to the burst neurons to zero (dashed line). Without reset by deep inhibitory neurons, the model exhibited continuing movement creep resulting in significant hypermetric saccades for larger target angles. With the inhibitory reset, the saccade is targeted precisely and the variation is significantly reduced. The inhibitory reset is thus not only a mechanism for resetting the saccade generation system, but contributes directly to the saccade dynamics.

## Discussion

With a large-scale spiking-neuron level model we can make use of specific neurophysiological constraints as well as system-level and behavioural data. We can observe the activity of both individual model neurons and the resulting large-scale behaviour. That, in turn, enables us to compare model behaviour with experimental data at both large-scale and fine-grained levels.

We show that when taking small-scale neurophysiological structure and large-scale behaviour into account, our resulting model is consistent with theories, such as [5], that assume that the superior colliculus plays an active part in driving saccadic eye movements.

Burst neurons driven by NMDA and cholinergic synaptic input exhibit sustained bursting, and an inhibitory feedback circuit between collicular burst neurons and a spike integrator circuit in the central mesencephalic reticular formation modulates the burst profile over time.

This study predicts that inhibition from the deep layer plays a role in stopping the eyes at the right time. This can be tested by selective manipulation of GABAergic neurons in the deep layer, which should be possible by using optogenetic methods. Moreover, the inhibitory neurons in the deep layer has been reported only in rabbits, so checking their existence in rodents and primates is also an important anatomical test.

The deep layer model neurons activate through mutual excitation; this mechanism is unrealistic but chosen for simplicity. NMDA receptor-based bursting or intrinsic bursting modulated through recurrent inhibition would be feasible mechanisms, but evidence for any mechanism is presently lacking.

A more specific prediction is that the balanced NMDA and inhibitory inputs as shown in Figure 4 is necessary for the proper peak speed and duration of the saccade. We predict that the rostral neurons have stronger NMDA inputs with associated stronger inhibitory inputs, which can be experimentally tested quantitatively by intracellular recording, or qualitatively by extracellular recording with pharmacological manipulations.

### Saccade main sequence

A straightforward relationship between saccade distance in collicular surface coordinates on one hand, and connection weights from buildup neurons and from a local spike integrator to burst neurons on the other, is sufficient to recreate the saccade target distance-related main sequence profile and peak skewness. With a theoretically derived distribution of weights from the SC to the motoneuron systems [47] and a static gain factor, we can approximate the resulting saccade.

Oblique saccades exhibit component-wise lower peak speed and longer duration than their purely horizontal or vertical counterparts [40]. Without component-wise stretching the end times would differ between the components resulting in curved saccades, and without adjustments to peak speed the saccade would overshoot its target. The components are thus non-separable and depend on each other.

The superior colliculus is the final common point for the horizontal and vertical eye motoneuron systems in our model. [40] argues that the saccade dynamics-generation circuitry is likely shared between horizontal and vertical systems due to the very small variations in initiation times between them, but that implies that the shared circuit needs to handle the component-wise variation to saccade angle. Our model oblique saccades show reduced peak speed and longer duration compared to horizontal saccades with the same horizontal component, as would be expected if the SC is to be the source of eye movement dynamics.

### Spreading activity

An open question concerns the functional significance of spreading activation seen in the intermediate superior colliculus. One suggestion is that the activity is tracking gaze direction error and that an end of saccade is triggered when the spreading activity reaches the collicular rostral pole [48]. However, evidence for this theory is weak or contradictory in the primate, as the spreading activation is too slow to track eye error and spreading activation never reaches the rostral pole for longer saccades [49]. [50] suggests the position error is encoded by decaying neuron activity in the monkey while still triggering the saccade end by activation of the rostral pole. [24] finds evidence against their role as a spatial integrator in the monkey but for a role in regulating the trajectory of each movement component. The detailed anatomical structures and functional characteristics of cat and primate SC shows significant differences (see [51] for an overview), so it is quite possible that the functional significance of spreading activation may differ between species as well. Most current theories have in common that they assign primary significance to the spatial structure of the activation, whether the front edge of the spread or the average activity position.

We suggest that spreading activation in the primate tracks saccade progression rather than gaze error; that total activation rather than the spatial spread is the significant measure; and that it terminates the saccade by activity level-induced deep neuron activation rather than rostral activation from the directional spread. In our view the spread is the means by which total buildup neuron activity increases over time.

We do not offer a functional explanation for the rostral shift in activity. The shift in primates is very weak for long saccades and might not have a significant functional role. In this work we have added a rostral shift to be in accord with the effect.

The connections between buildup and deep inhibitory neurons are uniform across the surface; the timing of the resulting saccade circuit termination is determined by the rate of spreading activation, which in turn is determined by the position-dependent interconnections in the burst neuron circuit. This termination not only prepares the system for a new saccade, but also directly improves the reliability of the burst generation circuit by regularizing the end of the burst envelope as seen in figure 7.

### Future directions

Human peak saccade speed tapers off sharply after about 20, and human saccade duration in figure 7B shows a steeper increase than the model output [45]. Both differences can be attributed to the fact that saccades beyond about 20 normally include head movements. Eye-only movement profile for larger saccades will be affected by a saturating eye motor plant. Inclusion of more realistic eye plant model and head movement is required for reproducing large angle saccades.

It is feasible to add rostral pole-mediated saccade inhibition and release to the model. Rostral fixation neurons poly-synaptically inhibit caudal SC burst neurons. Buildup neuron ramp-up would indirectly inhibit rostral fixation neurons that in turn releases the local inhibition and enable the release of the caudal saccade. The inhibition of burst and buildup neurons at the end of the saccade would in turn disinhibit rostral areas and re-enable caudal inhibition.

The present model lacks a true superficial division. We implement only the wide-field and narrow-field neurons that act as outputs to the intermediate areas, with no intraconnections; we also do not implement any input-specific intermediate circuits. Without a retinal model or a full superior division we had to simplify the visual input as a fixed, rather than transient, Poisson spike train, which we assume to be representative of the kind of input generated by the superficial division, the FEF and input-related intermediate circuits after regularization.

The model we have described here consists of only one superior colliculus. The lack of a interconnected pair implies that we can not correctly generate activity near the edges of the structure. The functional mapping between colliculi is fairly well understood [47], but how that mapping is realized among subpopulations of neurons in the superior colliculus is only beginning to become clear [52].

### Conclusion

This spiking neuron-level model generates collicular burst neuron activity through NMDA synapse mediated bursting and an inhibitory feedback circuit, and presents a functional mechanism for the spreading activation in the SC that tracks collicular activity and terminates the saccade via deep layer inhibitory neuron activation. The current model is able to account for recorded burst neuron output profiles as seen in figure 5, and the estimated eye movement behaviour recounted in figure 6 qualitatively reproduces the eye movement behaviour seen in biological systems.

## Materials and Methods

### Network structure

#### Neuron model

We use the Adaptive Exponential Integrate and Fire (AEIaF) neuron model by Brette and Gerstner [53] as implemented in the NEST simulator [54], and extend it with a membrane voltage-dependent NMDA synapse. The AEIaF model is a conductance-based integrate-and-fire model with an exponential soft spiking threshold and a second internal state variable 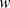 that recreates membrane potential- and spike-adaptation effects. Using this model we can deploy neurons with parameters and behaviour similar to corresponding real neurons. Table 2 summarizes the parameters used.

**Table 2:** AEIaF neuron parameters.

Parameter	Value	Parameter	Value
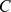	62/40 pF	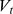	−47 mV
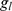	4 nS	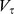	−65 mV
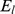	−65 mV	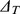	2.0 mV
**Adaptation**			
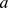	0.2 nS	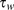	20 ms
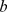	30 pA		
**Synapses**			
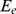	0.0 mV	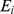	−75.0 mV
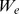	0.72 nS	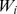	0.04 nS
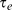	0.2 ms	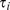	1.5 ms
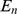	0.0 mV	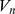	−51.8 mV
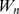	1.2 nS	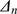	1.367
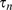	3.0 ms		

Parameters used for the AEIaF neuron for the simulation model. Burst neurons have a membrane capacitance of 40 pF.

The membrane potential follows the differential equation
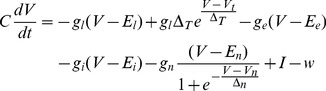
(1)where 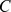 is the membrane capacitance, 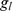 is the leak conductance, 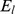 is the resting potential, and the exponential term creates a soft spiking threshold around 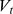 with softness determined by 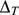. 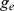, 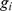 and 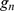 are the excitatory, inhibitory and NMDA synaptic conductances respectively, with synaptic reversal potentials 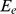, 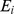 and 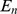.


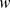
 is an adaptation current with time constant 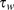 and sub-threshold adaptation level set by 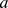:
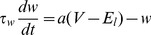
(2)


A spike event is triggered when the membrane potential diverges to infinity due to the exponential term; in practice a spike is triggered when 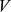 reaches 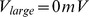. The membrane potential is reset to 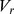 and a spike adaptation term 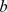 is added to the adaptation current 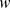:
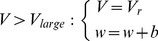
(3)


The synaptic conductances are shaped by an alpha function with the time 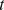 from the presynaptic spike and the time constant 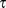:
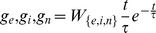
(4)


NMDA synapses are glutamatergic receptors sensitive to the membrane potential. We model an NMDA synapse as a sigmoidal function with center at 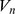 and gain 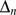 set so that curve approaches 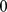 near 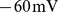 and 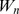 near 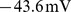. When multiplied by the voltage-dependent conductance 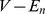, the resulting activation function will asymptotically approach the conductance gradient with peak activation at 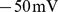.

The synthetic cMRF integrator is implemented as a set of 100 integrator units whose firing rate in spikes/s is linearly proportional to the sum of weighted spikes received since reset:
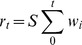
(5)where 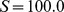 is the weighted total spikes that will elicit 1 spike/s. An inhibitory input will reset the integrator to zero.

### Synaptic connections

The synaptic connectivity parameters are summarized in table 1. The retinal mapping to the superior colliculus is log-polar in primates [47]. The mapping from retinal poplar coordinates 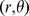 to the SC surface position 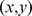 in mm is given by:
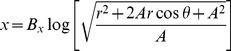
(6)

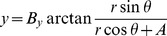
(7)where 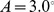, 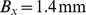 and 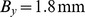 are parameters for monkey retina [55].

Inputs are mapped to the superficial neurons with projections with a standard deviation of 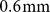 and a cutoff at 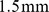 (table 1), convolved with the log-polar mapping in equation 6 and 7. The mapping width decreases caudally, but this is counterbalanced by the increase in retinal ganglion cell receptive field size to create a mapping that is roughly constant across the SC [38]. The resulting projection is asymmetric and rostrally shifted. The input is 100k spikes/s. While the model is sensitive to the projection size, the input rate can vary substantially without a significant effect on the model output.

The superficial neurons, QV, buildup and associated interneurons have a density of 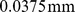 (about 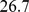 neurons per mm), for model layers of 128*147 neurons. Burst neurons and deep layer interneurons are relatively sparse [15], [56], so we give them half linear resolution (1/4 total neurons) for 64*73 units. The SC is not rectangular, so those units that fall outside the area are removed prior to the start of simulation.

Intraconnections in the model are Gaussian or flat (implemented by a wide Gaussian with cut-off):
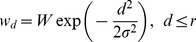
(8)where 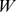 is the center weight, 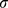 is the standard deviation and distance 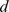 from the center is smaller than maximum radius 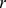.

Intraconnections in the intermediate model layers are all localized, less than 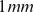, rather than long-range [36]. The buildup neuron and quasivisual neuron intraconnections, and the QV-buildup neuron interconnections are rostrally shifted.

### Eye movement model

The weights from burst neurons to the horizontal and vertical motoneuron system is given by [47]:
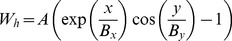
(9)

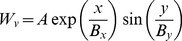
(10)where 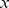 and 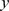 are the SC surface coordinates in mm. We use 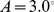, 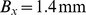 and 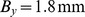 as above for the monkey retinotopic mapping.

We can obtain an estimate for the angular speed over time by determining the angular distance represented by each burst neuron spike.

The eye movement trajectory is estimated from the burst neuron output by accumulating the small eye displacement encoded by each spike, which is given by the horizontal and vertical angles (9) and (10) divided by the average total spikes (1485) for a saccade.

Collicular output is gain-adapted downstream of the burst neuron output [57], and we apply a constant gain to the output. The angular speed is the average rate multiplied by this distance.

To guide our model parameter estimation we assume that a saccade burst is over when the rate drops below 1% of the peak value. Due to the stochastic nature of our model we estimate the burst end in the simulations to be when activity drops below 10% of the peak rate.

[5] modeled the time course of burst neuron activation in the monkey as a saccade angle-dependent gamma-shaped function with fixed rise time as shown in figure 2. We use the formulation
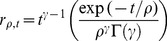
(11)

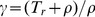
(12)where 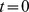 at the peak, 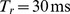 is the rise time and 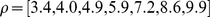 is an estimated angle-dependent time constant from [5]. Normalization and scaling with the peak neuron rate gives us
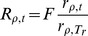
(13)where 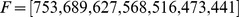 is the angle-dependent peak neuron burst rate, also from [5].
